# Inequality of Research Funding between Different Countries and Regions is a Serious Problem for Global Science

**DOI:** 10.1093/function/zqab060

**Published:** 2021-11-09

**Authors:** Ole H Petersen

**Affiliations:** School of Biosciences, Cardiff University, Cardiff, Wales CF10 3AX, UK

We are all increasingly conscious of the disparities with regard to opportunities for advancement in science between different ethnic groups and genders. Much is, rightly, being done to rectify this problem, although much more remains to be done. Meanwhile, there has been rather less concern about the vast inequalities in research funding between different countries and regions across the globe. It is clear that the global research effort is currently heavily concentrated in a relatively small part of the world, largely in North America, Europe, and in parts of the Far East, with relatively small contributions from Africa, large parts of Asia, and most of the Middle East. When I recently had the honour of giving the Gold Medal Lecture at Academia Europaea's Annual Conference in Barcelona,^1^ this problem was one of my principal themes.

At the beginning of the 20th century, Europe was at the centre of global science and Germany was the strongest science country in the world, as testified—for example—by the comparatively large number of Nobel Prizes given to German scientists. German science—and indeed most of the scientific research effort in Continental Europe—was destroyed, first by Nazi/Fascist politics in the 1930s and then by the Second World War. Unsurprisingly, many scientists from all over Europe decided to leave this part of the world, in which opportunities seemed to be very limited, and seek a more attractive way of life and work in the US. The European Brain Drain did much to develop scientific research in the US, which then rapidly became the world's most important science country.

European science has of course since then been remarkably revived and, certainly in the life sciences, is currently the second most important region in the world, after the US, with regard to the amount of high-quality scientific output. However, the European research enterprise is very unequally distributed across the region. Even within the European Union (EU) there are still today enormous disparities between research and development (R&D) funding in different countries. If one looks at government budget allocations for R&D per person in different EU countries, it can be seen that this allocation is more than 10 times higher in Denmark, Finland, and Germany than in Bulgaria, Hungary, and Romania.^2^ The research base is, therefore, much stronger in, for example, the Nordic countries than those in the Balkans and this has a major impact on the ability of researchers to win grants from EU funding bodies such as the European Research Council (ERC). Scientists in the poorly resourced European countries therefore face a double penalty; their own countries do not support science adequately and the probability of winning international grants is low. Unfortunately, this creates a vicious circle. Lack of funding makes it difficult to do ‘beyond state-of-the-art’ research or even just ‘state-of-the-art’ work and this limits opportunities for highly visible publications in top journals. This further reduces the chances of winning competitive international grants which, again, diminishes the research effort. The disparities in science funding between the relatively rich EU countries in the North-Western part of Europe and those in the East and South- East constitute, in my opinion, a serious problem. It is, in effect, holding back the whole of Europe, because we are unable to exploit all the great talents in this region. This matters in itself, considering the many and very considerable scientific and medical challenges we face, and also weakens Europe internationally. Ultimately, the prosperity and welfare of Europe, like all other parts of the world, depend on scientific knowledge and the ability to apply this knowledge.

Some might argue that it does not matter in which geographical location scientific work is being carried out as long as the results are available in the public domain, so that every country can benefit from the new knowledge. For example, the development of the novel mRNA vaccines against COVID-19 in Germany and the US^3^ have benefited the populations of many other countries. Nevertheless, a strong science base makes a country more powerful and influential as well as having major educational benefits locally. Scientific advice from a broad range of research-active experts to their local governments allows for more rational policy making, which is likely to further strengthen their performances. Countries with a strong science base also become particularly influential internationally with regard to the increasing number of issues that can only be solved by evidence-informed policy making. In the EU, for example, the Scientific Advice Mechanism relies on Evidence Review Reports produced for SAPEA (Science Advice for Policy by European Academies) by Working Groups composed of experts in the relevant fields.^4^ Countries with a strong science base will inevitably have a better chance of being represented on these Working Groups than those without such expertise and will therefore be more influential with regard to overall policy making.

There are also marked disparities in research funding between different regions in individual countries, with consequences similar to those outlined above. In the UK, for example, the governmental research council spend per capita is close to four times higher in England than in Wales.^5^

One of the members of the ERC Grant Panel I chaired a few years ago made the important observation that truly original ideas often come from ‘unusual’ places. This is an important argument for diversity in funding allocations, because if almost all research grants go to the ‘usual’ places, we shall mostly get more of the same but, of course, carried out with great competence. Although the stated philosophy of the ERC is to fund high-risk, high-gain projects, what really happens, in my experience, is that grants are mostly awarded to those who have already published in high-impact journals and who can, therefore, most convincingly persuade the assessors that their new research will continue to yield such publications. It seems to me that most funding bodies, whether they will admit it or not, actually prefer to play safe.

Free access to the published literature for everyone, irrespective of their location, is an essential, but not sufficient, condition for creating an equal global playing field. Open Access journals like *Function* fulfil an important role but, unfortunately, there are still today very many journals behind paywalls, causing significant problems for scientists in many parts of the world. Publishing is, of course, not cost-free. Someone has to pay and for Open Access journals, Article Processing Charges are, therefore, needed. Unfortunately, these can be a very significant burden for those with modest or no grant incomes. As a global community, we have still not come up with a fully functional and equitable funding system that would allow free-access publication from anywhere in the world, based solely on original ideas and quality of research, rather than ability to pay.

What is to be done to solve these problems? Rich countries could allocate a certain proportion of their research funding to support research in poor countries. This would help to limit the current movement of talented investigators from poor to rich countries; effectively a donation of a very valuable resource from poverty-stricken regions to the richest parts of the world. We also need to change the attitude of those sitting on international and national committees that make funding decisions. The, in practice, widespread adoption of the ‘winner takes all’ philosophy or the Matthew Principle (those who have shall have more and those who have little shall have even that taken away from them) needs to change. Too often, grants go to groups that already have massive funding, depriving researchers from less privileged places and backgrounds of even small research grants that could have made a real difference.
